# Do children in food-insecure households in England access free school meals? A cross-sectional analysis of the UK Family Resources Survey

**DOI:** 10.1136/bmjph-2025-004455

**Published:** 2026-09-18

**Authors:** Rosalyn Arnold, David Taylor-Robinson, Dougal Hargreaves, Rachel Loopstra

**Affiliations:** 1 Public Health, Policy and Systems, University of Liverpool, Liverpool, UK; 2 Imperial College London School of Public Health, London, UK

**Keywords:** Public Health, Cross-Sectional Studies, Food Security, Social Medicine

## Abstract

**Introduction:**

Food insecurity affects over one in four UK children. Free school meals (FSMs) may mitigate some adverse effects yet how well they reach food-insecure children is unclear. In September 2026, eligibility criteria will be changed but provision will not be universal. This study provides an analysis of FSM reach among food-insecure children and the potential impact of the 2026 eligibility changes.

**Methods:**

Data from the 2022–2023 and 2023–2024 Family Resources Survey were merged, yielding 6563 children aged 8–17 in England. Household food insecurity over 30 days was assessed using a validated measure, with marginal, low and very low food security classified as food-insecure. Weighted proportions of food-insecure children not receiving FSMs were estimated. Logistic regression identified characteristics associated with food insecurity among those not receiving FSMs and non-uptake among eligible children. Projected impacts of 2026 eligibility changes were estimated under two scenarios.

**Results:**

70.6% (95% CI 68.0% to 73.2%) of food-insecure children did not receive FSMs. Among these, 80.2% (95% CI 77.5% to 82.9%) were ineligible, while 19.8% (95% CI 17.1% to 22.5%) were eligible but not taking them up. Household characteristics associated with food insecurity included black/African/Caribbean ethnicity (aOR: 2.37, 95% CI 1.64 to 3.42) and disabled members (aOR: 1.71, 95% CI 1.41 to 2.06). Non-uptake was more common among older pupils (aOR per year: 1.19, 95% CI 1.11 to 1.28) and households without disability (aOR: 3.53, 95% CI 1.91 to 6.51). In modelled scenarios, over 270 000 children may gain eligibility in 2026, yet 44.5% (95% CI 41.7% to 47.2%) of food-insecure children could remain ineligible.

**Conclusions:**

2026 FSM eligibility changes are likely to reach more food-insecure children, but many may remain ineligible, particularly those facing structural disadvantage. These findings highlight the limitations of benefit-based criteria. Universal FSM provision has been proposed as a more equitable approach, with the potential to ensure that all food-insecure children are reached.

**WHAT IS ALREADY KNOWN ON THIS TOPIC**

- Research from the UK, USA and Europe demonstrates the positive effects of free school meals (FSMs) on children’s health, well-being and educational outcomes. In England, however, FSM eligibility, which is tied to benefit receipt and income thresholds, excludes many households that are below low-income measures.

**WHAT THIS STUDY ADDS**

- This study provides the first national estimates of FSM reach among food-insecure children in England and assesses how September 2026 eligibility changes may affect this. Each year between 2022 and 2024, over 1.1 million children in food-insecure households were not receiving FSMs, highlighting the disconnect between income-based eligibility and lived experiences of food insecurity. Projections show that while 2026 eligibility changes are likely to increase reach, 44.5% of food-insecure children may remain or become ineligible.

**HOW THIS STUDY MIGHT AFFECT RESEARCH, PRACTICE OR POLICY**

- Findings support the 2026 eligibility change but highlight the limitations of continuing to use benefit-based criteria in determining eligibility. Incremental changes, such as national auto-enrolment, could improve reach; however, universal FSM provision has been proposed in wider literature as a more equitable approach for ensuring that all food-insecure children.

## Introduction

 Food insecurity, defined as ‘the inability of households to afford sufficient food’,^1^ has been monitored in the UK by the Department for Work and Pensions (DWP) since 2019. In 2023–2024, 10% of households experienced food insecurity each month, rising to 16% when marginal food security was included.^2^ Alarmingly, prevalence has increased in recent years, particularly among households with children. The most recent data from the DWP showed that 25% of all households with children reported marginal, low or very low food security in the past month, a rise of 6 percentage points since 2019–2020.^2,3^ This represents one million more children experiencing food insecurity each month than in 2019–2020.

Children in food-insecure households, even those classified as marginally insecure, face a range of adverse outcomes. Evidence links food insecurity with delayed language and social development, lower academic performance, poorer mental health, social exclusion and stigma, and worse physical health.^4–12^ The latter includes poor growth, underweight, vitamin and mineral deficiencies, lowered immunity, more frequent hospitalisations and higher risk of chronic diseases such as obesity and diabetes.^6,7,9,10,12^ Interventions to protect children against these adverse outcomes are critical.

Free school meals (FSMs) are one potential effective intervention. International evidence shows that FSM provision improves both health and educational outcomes. In the USA, access to free or reduced-price school meals through the National School Lunch Programme has been linked to reduced food insecurity, improved health and lower obesity.^13,14^ In Norway, a randomised controlled trial demonstrated that providing a daily healthy FSM for 1 year increased children’s consumption of nutritious foods compared with children bringing in a packed lunch, particularly among children from low-income families.^15^ In the UK, qualitative research suggests FSMs protect children against some of the harms of food insecurity and reduce parental stress about providing lunches.^5,16–18^ Together, these studies provide strong evidence that FSMs play a protective role against some adverse outcomes associated with food insecurity.

Across the UK, USA and Europe, there is a mix of universal and means-tested provision. Universal provision may yield stronger benefits. In Sweden, where FSMs have been universally available to all primary pupils since 1969, an evaluation found immediate improvements in educational attainment and health, as well as a 3% increase in lifetime income, with the greatest benefits for low-income families.^19^ In the USA, schools offering universal FSMs reported reduced food insecurity, higher attendance, reduced stigma, increased meal participation and improved academic performance compared with schools offering means-tested provision.^20–25^ Where universal provision has been implemented in England, evidence suggests that, compared with means-tested programmes, universal FSMs are easier to implement and lead to higher uptake and educational attainment among lower-income families, as well as lower rates of obesity.^26–29^

In England, the current policy offers universal infant FSMs for children in reception to year 2 (covering ages 4–7). From year 3 onwards, eligibility is restricted to children from families receiving income-replacement benefits (see table 1). Income thresholds apply to some benefits. Children have also been eligible under ‘transitional protections’ since 2018, which allowed once-eligible children to remain eligible until September 2026 or until their current education phase ended (ie, a move from primary to secondary school, for instance), even if their family’s circumstances no longer met eligibility criteria. In most local authorities, households must also complete and submit an application to receive FSMs.

**Table 1:** Eligibility for free school meals in England for households with pupils in maintained schools, academies and free schools from year 3

Group	April 2018 to August 2026^50^	September 2026 forward^34^
Children in household with parent/guardian who claims Universal Credit	Eligible only if an annual net earned income of no more than £7400, as assessed by earnings from up to three of the most recent assessment periods	All will be eligible
Children in household with parent/guardian who receives the following legacy benefits: Income support Income-based jobseeker’s allowance Income-related employment and support allowance Child Tax Credit (provided not also entitled to Working Tax Credit and have an annual gross income of no more than £16 190) Working Tax Credit run-on—paid for 4 weeks after stopping to qualify for Working Tax Credit	Eligible	All legacy benefit claims will have ended, so unless a new claim for UC is made, children will become ineligible. Transitional protections no longer apply
Children in household covered by transitional protection (ie, first eligible for FSM due to UC claim, but circumstances changed to make no longer eligible)	Eligible	No longer eligible Transitional protections no longer apply
Children in household that receives Child Benefit	Not eligible unless eligible by criteria above	Not eligible unless eligible by criteria above
Children in household that receives personal independence payments	Not eligible unless eligible by criteria above	Not eligible unless eligible by criteria above

Universal Credit was rolled out in the UK from 2013. It replaced six income-replacement benefits, referred to as ‘legacy benefits’. No new claims for legacy benefits could be made from December 2018 and by March 2026, all legacy claimants should be moved to UC. Additional eligibility for FSM includes support under Part VI of the Immigration and Asylum Act 1999 and the guaranteed element of Pension Credit. To our knowledge, these eligibility criteria are not changing.

FSM, free school meal; UC, Universal Credit.

Even with transitional protections in place, concerns have long been raised that eligibility criteria are too restrictive, with estimates suggesting that many low-income families do not qualify because they are not in receipt of qualifying benefits or have incomes above specified thresholds.^30^ In addition, the application process can be onerous.^31^ Organisations have advocated for universal provision to reduce stigma and improve uptake.^32,33^

In June 2025, the UK Government announced that, from September 2026, eligibility will be extended to all children in households receiving Universal Credit (UC), abolishing current income thresholds.^34^ However, this policy coincides with the national closure of all remaining legacy benefit claims in August 2026 permanently.^35^ Families that do not transition to UC, or that qualified through transitional protections but no longer meet eligibility criteria, will no longer be eligible for FSMs (table 1). While the extension of FSMs to all UC recipients has been welcomed, charities warn that children in households yet to migrate to UC risk losing access.^36–39^ Taken together, these changes mean a widening in eligibility for some families but potential exclusion for others.

To date, no studies have examined the reach of FSMs in relation to food insecurity under the eligibility criteria in place in England until September 2026, nor has the potential impact of the new criteria been assessed, though government estimates claim that 100 000 children will be lifted out of income poverty as a result of the extension.^40^ Analysis from the Child Poverty Action Group indicated that in 2023–2024, 900 000 children in income-poverty were not eligible for FSMs.^30^ Both estimates, however, are grounded in income-based definitions of poverty and do not account for children who are eligible for FSMs but do not take up provision.

Given ongoing debates over universal provision, it is also important to assess whether government criteria for FSMs identify families vulnerable to food insecurity and to identify groups at risk not covered by current criteria. FSM eligibility is an administrative construct based on benefit receipt and income thresholds, whereas food insecurity reflects a lived experience shaped not only by low income but also by debt, rising living costs and financial insecurity.^6,8^ Misalignment between the two may therefore be expected. The extent to which this administrative construct effectively identifies and reaches households experiencing food insecurity is under-examined. Recent policies in Wales, Scotland and London that expand FSM access to all primary pupils underscore the importance of evaluating England’s current system and the implications of forthcoming changes.^41–43^

This study addresses these gaps. We aim to quantify the number of children aged 8–17 years living in food-insecure households in England who did not receive FSMs. We then examine whether this is driven primarily by ineligibility based on current benefit and income criteria or by non-uptake. We identify characteristics associated with food insecurity among children not receiving FSMs and compare FSM-eligible children in food-insecure households who do and do not take up provision. Finally, we estimate how FSM coverage among children in food-insecure households may be affected by changes to FSM eligibility from September 2026.

## Methods

### Dataset

Data from the Family Resources Survey (FRS), an annual cross-sectional survey of UK private households, was used. In the main analysis, data from the 2022–2023^44^ and 2023–2024 FRS^45^ were merged. These datasets incorporate linked administrative benefit records to improve the recording of benefit receipt.^46^ These updated releases replace the original survey datasets and were released in May 2026. In 2023–2024, the FRS sampled 16 754 households, of which 4294 contained children, representing 7658 dependent children aged 0–18. In 2022–2023, 25 045 households were surveyed, of which 6238 contained children, representing 11 097 children.

The FRS collects different data at three levels: household, benefit unit (a single adult or couple, plus any dependent children) and individual. Below we describe how variables from household, benefit and child datasets were combined for analyses.

### Variables

#### Food insecurity

Food insecurity was measured at the household level using the US Department of Agriculture (USDA) 10-item Adult Food Security Survey Module, with a 30-day reference period.^47^ Questions were answered by the adult household member with knowledge of food acquisition and preparation. Households were classified as having high, marginal, low or very low food security based on the number of affirmative responses to the module. For this study, households reporting marginal, low or very low food security were classed as food-insecure, as were children living in these households, while those reporting no affirmative responses were classified as food secure. UK governmental figures typically classify food insecurity as those experiencing low and very low food security. We adopted a broader classification to include marginal insecurity, reflecting evidence that it too is linked with adverse outcomes.^48,49^ This ensures our analysis captures all children at risk. Sensitivity analyses using the UK Government threshold were also conducted.

#### Receiving FSM

FSM receipt was recorded at the individual child level, based on the primary carer’s report of whether each child had received at least one FSM in the past week. This was a binary variable. Since children may differentially receive FSMs within households, receipt of FSMs was examined at the individual child level.

#### Current FSM eligibility

FSM eligibility is not directly measured in the FRS. We derived it at the benefit unit level using Government guidelines from the 2023 to 2024 academic year.^50^ Children were considered eligible if, at the time of the survey, their benefit unit received UC (provided annual net earned income is below £7400), Income Support, Job Seekers Allowance, Employment Support Allowance, guaranteed element of Pension Credit or Child Tax Credits (provided not also in receipt of Working Tax Credit and annual gross income is below £16 190) (table 1). Of note is that we could only derive eligibility based on administrative benefit receipt and income criteria. Other routes to FSM entitlement, such as transitional protections, could not be identified. A flow chart illustrating the derivation of FSM eligibility is provided in online supplemental web appendix 1.

The FRS records detailed income data from multiple sources, which are validated against documentation where possible. Incomes are reported as weekly amounts and then converted to annual net earned income to align with FSM eligibility thresholds.

#### Post-September 2026 FSM eligibility

New FSM eligibility was derived from the Government’s guidance for September 2026 onwards.^34^ Children were considered eligible from September 2026 if their benefit unit was in receipt of UC. To capture the impact of ending transitional protections, we assumed that children receiving FSMs but not meeting current derived eligibility criteria were covered through transitional protection.

To capture the potential impact of the closure of legacy benefits, we used data from the DWP which suggests that around 27% of households sent a notice to move to UC do not do so.^51^ It is important to note that this transition rate is based on incomplete data, does not represent all legacy benefit claimants, and could change, but it was the only rate available at the time of analysis.

#### Characteristics associated with FSM non-uptake and food insecurity

Ethnicity, region, deprivation, parental employment and education have been found to be associated with non-uptake of FSMs.^52,53^ Risk factors for food insecurity included ethnicity, region, household size, disability, income, benefit receipt, lone parent status and parental education.^6,54–59^ We included these variables in our model, as well as a variable denoting current FSM eligibility to test whether the current policy criteria identify children at risk. Child age was included given ongoing debates about whether FSMs should be extended to all primary or secondary pupils.

For this analysis, income was categorised according to the annual UC FSM income threshold (£7400), above this but below Northern Ireland’s UC FSM threshold (£15 000)^41^ and the 2024 Joseph Rowntree Foundation’s minimum income standard for a two-adult, two-child household (£66 200).^60^

### Analytical samples

Our analytical sample only includes children in England due to differences in FSM eligibility between devolved nations. In addition, observations from August were excluded as children were not in school during this month. Children in primary schools in London during the 2023–2024 school year were removed as they were eligible under the Greater London Authority’s (GLA) primary universal provision. A complete case analysis was conducted, excluding households with missing data for outcome variables and predictor variables of interest, as well as cases with conflicting or ambiguous information on key variables (n=43).

After exclusions, our analytical sample was 4410 households with children aged 8–17 in full-time education in England, representing 6563 children (see online supplemental web appendix 2). These children were not covered by the Universal Infant FSM policy or the GLA expansion to all primary pupils in 2023–2024.

### Statistical analysis

First, we calculated the number of food-insecure children who did not receive FSMs between 2022 and 2024. We then assessed whether non-uptake of FSMs among food-insecure children was driven primarily by ineligibility or non-uptake by classifying children as eligible or ineligible according to benefit receipt and income threshold criteria. Weighted proportions and national estimates were derived using FRS survey weights, which adjust for non-response bias and gross the data to the national population. CIs were estimated using the ‘*survey*’ package in R.^61^

To identify characteristics associated with food insecurity among children not receiving FSMs, logistic regression models were fitted at the household level, reflecting the level at which food insecurity is measured. Child age was represented by household-level dummy variables for the presence of primary or secondary age children. A series of nested models were conducted to assess the implications of expanding FSM eligibility under proposed eligibility changes, allowing examination of how alternative criteria and household characteristics relate to food insecurity. The initial model included only current eligibility based on income and benefit receipt. We then added receipt of UC, capturing those qualifying under expanded 2026 criteria. Legacy benefit claimants were added separately because they will either have to transition to UC under the new criteria or will no longer be eligible. Then we introduced child and disability benefits not included in UC to assess whether broadening eligibility to these benefit recipients might extend reach to food-insecure households outside eligibility criteria. Finally, other risk factors for food insecurity were added to explore vulnerability beyond benefit receipt.

To identify characteristics associated with non-uptake, we examined the risk of not receiving FSMs among food-insecure children eligible for FSMs. FSM uptake was measured at the individual child level as this is the level at which it is reported. SEs were clustered at the household level.

Multicollinearity in both fully adjusted models was assessed using generalised variance inflation factors (GVIFs).

Our final analysis explored how FSM eligibility for food-insecure children may change under the policy which came into force in September 2026. Given the uncertainty around how many households will transition from legacy benefits to UC, we modelled two scenarios to project the effect of the closure of legacy benefits on FSM eligibility: (1) all households currently on legacy benefits successfully claim UC and remain eligible and (2) 27% of households on legacy benefits do not transition to UC, based on DWP estimates, and lose eligibility.^51^ To estimate the impact of ending transitional protections, we assumed that children reported as receiving FSMs despite not meeting derived eligibility criteria were covered through transitional protections. Both scenarios assume no changes in household composition, income or eligibility status between the survey period and policy implementation in 2026. These projections are conditional on the assumptions underlying each scenario and are intended to illustrate potential policy impacts rather than predict future outcomes.

All analysis was conducted using RStudio (V.2024.12.0+467).^62^

### Sensitivity analyses

We examined how results changed when we classified food insecurity based on low and very low food security, excluding marginal food security.

## Results

There were 6563 children aged 8–17 years in full-time education in the sample. At the time of the surveys, 13.5% (95% CI 12.5% to 14.4%) had received FSMs in the past week and 14.6% (95% CI 13.6% to 15.6%) were eligible according to the derived criteria used in this study. Overall, 52.9% (95% CI 49.2% to 56.6%) of eligible children were not taking up FSMs. Among children who were receiving FSMs, 42.8% (95% CI 38.9% to 46.7%) were classed as ineligible based on the derived criteria applied in this study.

**Figure 1:**
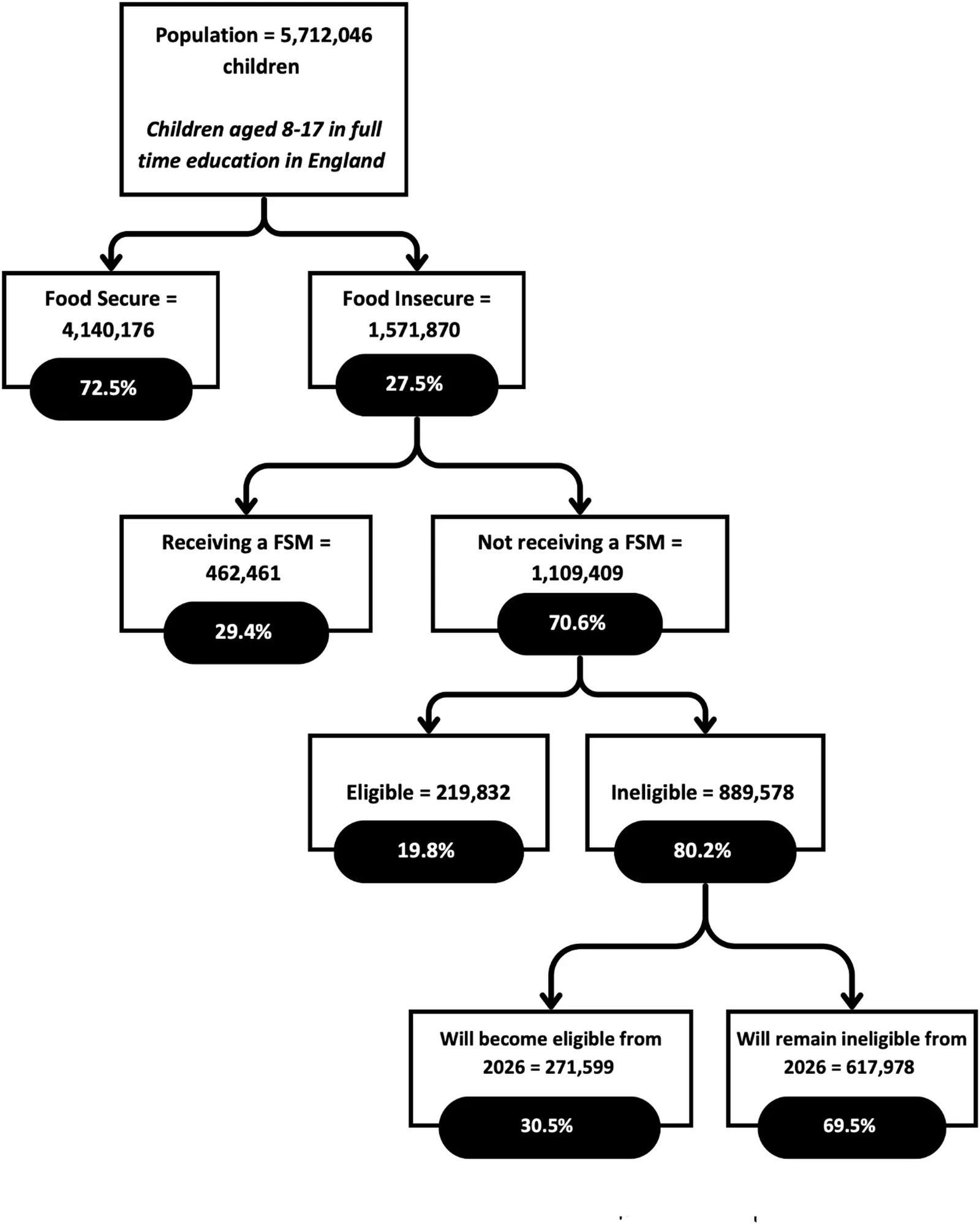
Flowchart illustrating the distribution of food insecurity, free school meal (FSM) receipt and eligibility across the population sample between 2022 and 2024, averaged to produce yearly estimates. See online supplemental appendix 3 for accompanying confidence intervals. Eligibility was classified using the derived eligibility criteria described in the Methods section, rather than observed administrative eligibility.

Of the 6563 children, 27.5% (95% CI 26.2% to 28.8%) overall were food-insecure. Among food-insecure children, only 29.4% (95% CI 26.8% to 32.0%) received FSMs (figure 1, online supplemental web appendix 3). Averaged across the 2 years, this equates to 1 109 409 children annually (95% CI 1 038 881 to 1 179 937).

Among food-insecure children not receiving FSMs, 80.2% (95% CI 77.5% to 82.9%) appeared ineligible under current income and benefit eligibility criteria, while 19.8% (95% CI 17.1% to 22.5%) appeared eligible but not taking FSMs up (figure 1, online supplemental web appendix 3).

### Characteristics associated with food insecurity among children not receiving FSMs

Household characteristics associated with food insecurity among children not receiving FSMs were examined (table 2, online supplemental appendix 4). Derived current FSM eligibility was initially significantly associated, but lost significance after adjustment for benefit receipt and other risk factors for food insecurity (model 3, table 2). Receipt of UC or legacy benefits remained strongly and independently associated across all models.

**Table 2:** Odds of food insecurity among households where at least one child is not receiving FSMs (n=3784)

	Odds of food insecurity (OR (lower CI, upper CI))			
	Base model (food insecurity~FSM eligibility)	Model 1: base model+Universal Credit and legacy benefits	Model 2: base model+all benefits	Model 3: base model+all benefits+other predictors
Current FSM eligibility* (*Ref=not eligible*)	**5.97 (4.65 to 7.65)**	**1.86 (1.42 to 2.44)**	**1.74 (1.3 to 2.31)**	1.21 (0.75 to 1.95)
Receipt of benefits	–			
No benefits		Reference category	Reference category	Reference category
Legacy benefits†		**5.72 (4.54 to 7.2)**	**5.6 (4.42 to 7.1)**	**2.49 (1.86 to 3.35)**
Universal Credit		**6.27 (5.08 to 7.75)**	**6.22 (5 to 7.74)**	**2.78 (2.11 to 3.66)**
Receipt of Disability Benefits‡ (*Ref=not in receipt*)	–	–	1.31 (0.93 to 1.86)	1.19 (0.82 to 1.73)
Receipt of Child Benefits (*Ref=not in receipt*)	–	–	1.02 (0.77 to 1.35)	1.02 (0.75 to 1.38)
Annual earned family income	–	–	–	
Under income threshold				Reference category
£7400–£10 999				1.27 (0.74 to 2.18)
£11 000–£14 999				0.89 (0.53 to 1.48)
£15 000–£25 999				0.83 (0.51 to 1.34)
£26 000–£39 999				0.92 (0.56 to 1.52)
£40 000–£66 199				0.62 (0.37 to 1.03)
£66 200 or higher				**0.24 (0.13 to 0.44)**
Ethnicity of first parent	–	–	–	
White				Reference category
Asian/Asian British				1.28 (0.95 to 1.72)
Black/African/Caribbean/Black British				**2.37 (1.64 to 3.42)**
Mixed/ Multiple ethnic groups				0.86 (0.45 to 1.66)
Other ethnic group				1.35 (0.78 to 2.32)
Highest education level in household	–	–	–	
Higher degree of postgraduate qualifications				Reference category
Undergraduate degree and equivalent				1.31 (0.97 to 1.79)
Diplomas in higher education and equivalent				**1.9 (1.32 to 2.75)**
A/AS levels and equivalent				**2.04 (1.48 to 2.83)**
O level/GCSE grades A–C and equivalent				**2.33 (1.61 to 3.38)**
O level/GCSE grades D–G and equivalent				**2.43 (1.61 to 3.67)**
No answer provided				**3.1 (2.01 to 4.79)**
Disabled individuals in household (*Ref=no disabled individuals*)	–	–	–	**1.71 (1.41 to 2.06)**
Household full-time employment (*Ref=no individuals in full-time employment*)	–	–	–	0.97 (0.72 to 1.3)
Unemployment in household (*Ref=no individuals unemployed*)	–	–	–	**2.01 (1.3 to 3.08)**
Lone parent household (*Ref=no*)	–	–	–	**1.33 (1.05 to 1.69)**
Region	–	–	–	
South				Reference category
London				**1.37 (1.02 to 1.83)**
North				1.08 (0.88 to 1.31)
Number of children, *per additional child*	–	–	–	**1.11 (1 to 1.24)**
Children in primary school (*Ref=no children in primary school*)	–	–	–	1.18 (0.92 to 1.51)
Children in secondary school (*Ref=no children in secondary school*)	–	–	–	1.07 (0.82 to 1.39)

To see all reference categories in detail, see online supplemental web appendix 4.

Bold estimates indicate statistical significance at 0.05 (95% CI does not include the null value of 1).

* Eligibility was classified using the derived eligibility criteria described in the Methods section, rather than observed administrative eligibility.

† Legacy benefits include housing benefits; working tax credits, child tax credits, income support, job seekers allowance, employment and support allowance and pension credit.

‡ Disability benefits include personal independence payment, adult disability payment, disability living allowance or child disability payment. Bold font indicates results that are statistically significant (p<0.05).

FSM, free school meal; UC, Universal Credit.

Households that appeared to have increased risk of food insecurity independent of current or proposed eligibility were those with a household reference person who identified as Black/African/Caribbean/Black British, those with more children, those with lower levels of household educational attainment, households based in London and those with disabled or unemployed household members (model 3; table 2). Income appeared protective only at the highest level. GVIF values were all under 2 in the fully adjusted model.

### Characteristics associated with non-uptake of FSMs among eligible food-insecure children

Next, characteristics associated with non-uptake among food-insecure children classified as eligible according to the derived eligibility criteria were examined (table 3). In bivariate analyses, older age and absence of disability in the household were associated with non-uptake, and these associations remained in the multivariate model. The odds of non-uptake increased by 1.19 (95% CI 1.11 to 1.28) for each additional year of the child’s age. A disabled household member reduced the odds of non-uptake (adjusted OR: 0.28, 95% CI 0.15 to 0.52). GVIF values were low (<2) in the fully adjusted model.

**Table 3:** Odds of not receiving an FSM among children currently eligible* and living in food-insecure households (n=553)

	Odds of not receiving free school meals (OR (lower CI, upper CI)	
	**Bivariate model**	**Multivariate model**
Age, *per year increase*	**1.16 (1.09 to 1.24)**	**1.19 (1.11 to 1.28)**
Region		
South	*Reference category*	*Reference category*
London	1.07 (0.58 to 1.98)	1.07 (0.55 to 2.09)
North	0.66 (0.42 to 1.05)	0.62 (0.38 to 1.02)
Lone parent household		
No	*Reference category*	*Reference category*
Yes	0.82 (0.53 to 1.29)	0.93 (0.56 to 1.54)
Ethnicity of first parent		
White ethnicity	*Reference category*	*Reference category*
Minoritised ethnicity	1.01 (0.6 to 1.69)	0.61 (0.33 to 1.11)
Receipt of benefits		
Legacy benefits†	*Reference category*	*Reference category*
Universal Credit	1.16 (0.72 to 1.85)	1.08 (0.63 to 1.86)
Receipt of Disability Benefits*		
Not in receipt	*Reference category*	*Reference category*
In receipt	1.22 (0.77 to 1.92)	1.55 (0.9 to 2.67)
Receipt of Child Benefits		
Not in receipt	*Reference category*	*Reference category*
In receipt	0.86 (0.26 to 2.8)	0.93 (0.31 to 2.75)
Number of children*, per additional child*	0.87 (0.73 to 1.04)	0.99 (0.82 to 1.21)
Highest education level in household		
Undergraduate or higher degree of postgraduate qualifications	1.97 (0.94 to 4.13)	1.98 (0.92 to 4.28)
Diplomas in higher education and equivalent	1.43 (0.6 to 3.37)	1.32 (0.5 to 3.47)
A/AS levels and equivalent	*Reference category*	*Reference category*
O level/GCSE and equivalent	0.69 (0.4 to 1.19)	0.7 (0.39 to 1.25)
Not answered/don’t know/prefer not to say	1.21 (0.63 to 2.33)	1.3 (0.64 to 2.66)
Disabled individuals in household		
No disabled individuals	*Reference category*	*Reference category*
At least one disabled individual	**0.41 (0.24 to 0.69)**	**0.28 (0.15 to 0.52)**
Household full-time employment		
No individuals in full-time employment	*Reference category*	*Reference category*
At least one individual in full-time employment	1.82 (0.9 to 3.68)	1.36 (0.61 to 3.06)
Unemployment in household		
No individuals unemployed	*Reference category*	*Reference category*
At least one individual unemployed	1.35 (0.7 to 2.63)	1.09 (0.53 to 2.28)

Bold estimates indicate statistical significance at 0.05 (95% CI does not include the null value of 1).

* Eligibility was classified using the derived eligibility criteria described in the Methods section, rather than observed administrative eligibility.

† Legacy benefits include housing benefits; working tax credits, child tax credits, income support, jobseeker’s allowance, employment and support allowance and pension credit.

‡ Disability benefits include personal independence payment, adult disability payment, disability living allowance or child.

FSM, free school meal; UC, Universal Credit.

### Projected effects of the 2026 eligibility changes

**Figure 2:**
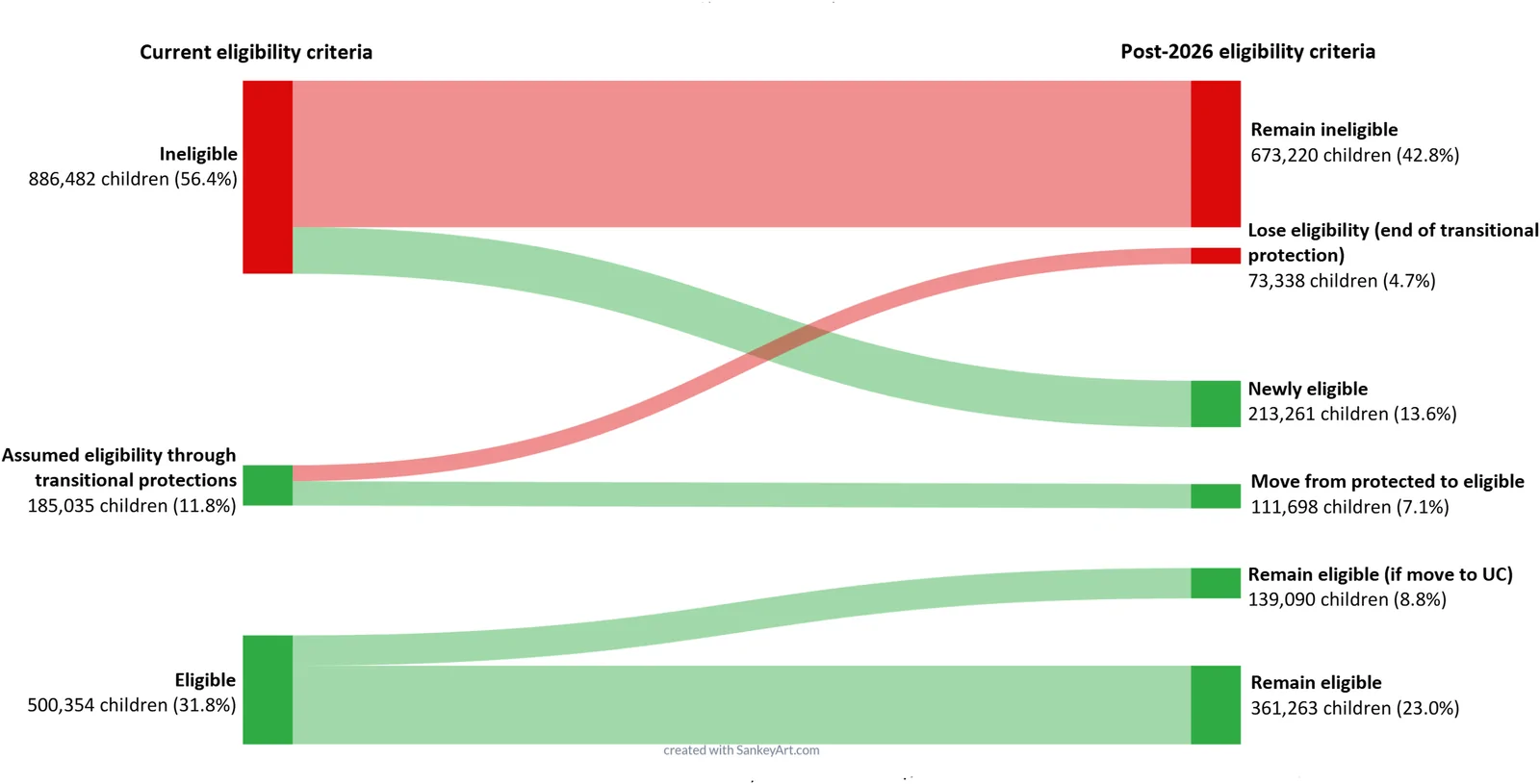
Proportionate Sankey diagram showing the forecasted annual changes in free school meal eligibility among food-insecure children (n=1 571 870) following September 2026 eligibility changes under scenario 1, which assumes all households successfully transition to Universal Credit (UC) and remain eligible. Percentages represent the proportion of all children in food-insecure households present in each category. Numbers are annual estimates. Results are scenario-based projections, conditional on the assumptions outlined in the Methods section, rather than predictions. CIs are provided in online supplemental appendix 5a.

In scenario 1 (figure 2, online supplemental web appendix 5a), where all households on legacy benefits successfully transition to UC, 17.3% (95% CI 15.1% to 19.4%) of all food-insecure children may gain eligibility in September 2026. However, 2.6% (95% CI 1.8% to 3.4%), equivalent to 40 869 children (95% CI 28 337 to 53 401), could lose access due to the ending of transitional protections. In this scenario, 58.1% (95% CI 55.3% to 60.9%) of food-insecure children could remain or become eligible, leaving 41.9% ineligible (95% CI 39.1% to 44.7%).

**Figure 3:**
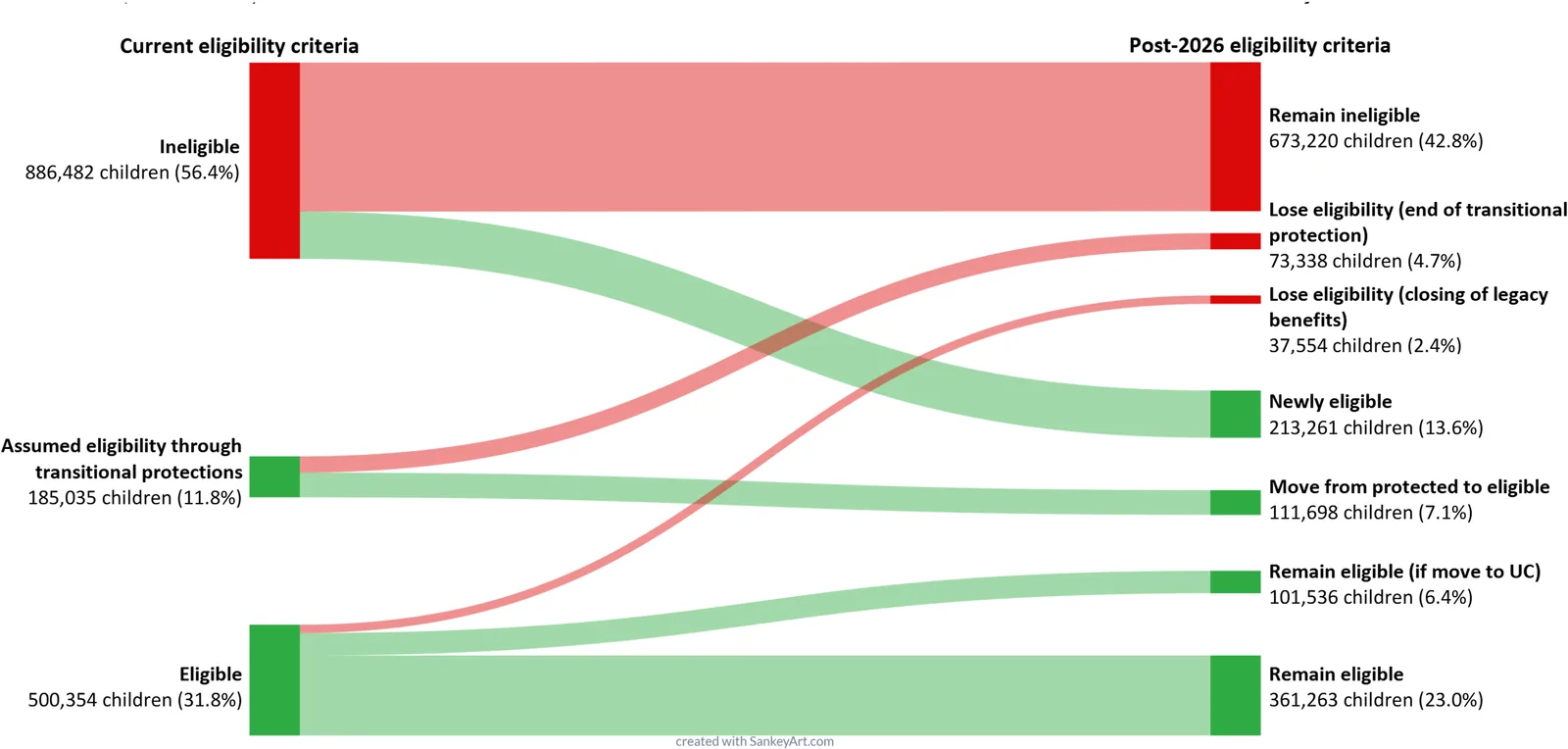
Proportionate Sankey diagram showing the forecasted annual changes in free school meal eligibility among food-insecure children (n=1 571 870) following September 2026 eligibility changes under scenario 2, which assumes 27% of households on legacy benefits do not transition to Universal Credit (UC).^51^ Percentages represent the proportion of all children in food-insecure households present in each category. Numbers are annual estimates. Results are scenario-based projections, conditional on the assumptions outlined in the Methods section, rather than predictions. CIs are provided in online supplemental appendix 5b.

In scenario 2 (figure 3, online supplemental web appendix 5b), where 27% of households do not transition to UC, a further 2.6% (95% CI 1.8% to 3.4%) of food-insecure children could lose eligibility. Combined with the loss of transitional protections, 5.2% of food-insecure children could lose FSM access overall. Under this scenario, 44.5% (95% CI 41.7% to 47.2%) of food-insecure children could remain or become ineligible.

### Sensitivity analyses

Results were broadly consistent when classification of food insecurity was limited to low or very low food security. In terms of proportions, 65.3% (95% CI 61.8% to 68.8%) of children in low/very low food-secure households were not receiving FSMs, compared with 70.6% of food-insecure children, including marginally food secure. In absolute numbers, this was estimated to be 680 798 (95% CI 620 614 to 740 984) children. A slightly smaller proportion, 74.9% (95% CI 71.0% to 78.7%), appeared ineligible compared with the analysis that included marginally food-insecure children (online supplemental appendix 6).

The fully adjusted logistic regression analysis examining low/very low food security among children not receiving FSMs showed the same variables to be associated with food insecurity, with the exception of unemployment, lone parenthood and living in London. Having an annual income above £40 000 was protective of this more severe threshold of food insecurity, whereas this was not found when marginal food insecurity was included. In the analysis examining non-uptake of FSMs among eligible children with low/very low food security, living in a minoritised ethnicity household reduced the risk of non-uptake (OR: 0.45 (95% CI 0.23 to 0.84), although low numbers mean this finding should be interpreted with caution.

Projected effects of the future 2026 eligibility under the restrictive classification estimated that 37.9% (95% CI 34.4% to 41.3%) of children in low/very low food-secure households were projected to remain or become ineligible under the 2026 changes in scenario 2. Full sensitivity analysis results are presented in online supplemental appendices 6–9.

## Discussion

This study provides the first detailed quantitative analysis of food-insecure children not receiving FSMs in England. Between 2022 and 2024, more than one quarter of children aged 8–17 years lived in food-insecure households. However, fewer than one in three of these children received FSMs, leaving over 1.1 million food-insecure children without this support each year. Even when restricting to households experiencing low/very low food security, the number of children in these households who were not receiving FSMs remained high.

Among food-insecure children not receiving FSMs, the majority were ineligible according to the derived eligibility criteria, yet around one in five were classified as eligible but not taking them up. This highlights the dual challenges of restrictive eligibility and barriers to uptake. Tackling both is required to ensure food-insecure children are able to access FSMs.

While current FSM eligibility criteria were initially associated with food insecurity, it lost significance in the fully adjusted model. This suggests that, while current eligibility correctly identified vulnerable children, income thresholds were too restrictive. Any receipt of UC or legacy benefits was strongly and independently associated, even after adjusting for income. This supports the change to make all UC-recipient households, regardless of income, eligible for FSMs. Though some of these children could have been eligible through transitional protections, this policy is being removed, and families may not have known they remained eligible. Our findings also suggest support must be given to ensure legacy benefit recipients successfully transition to UC.

Income was protective only at the highest level, with households earning above £66 200 less likely to experience food insecurity. This aligns with evidence that food insecurity is not restricted to the lowest income groups but is also influenced by factors such as income stability, access to savings and debt.^6,8^

Structural inequalities were also apparent, with children with a household reference person identifying as black/African/Caribbean, those with a disabled or unemployed household member, and those with lower educational attainment in household being at increased risk. These findings are indicative of broader structural inequalities in exposure to food insecurity and are consistent with a wide body of evidence showing that food insecurity is shaped by inequalities that extend beyond income alone.^54–59,63–65^ Persistence of these associations after adjustment suggests that current and post-2026 eligibility criteria, which are income- and benefit-based, do not fully capture underlying social and economic disadvantage and highlights the limitations of using benefit-based eligibility.

Within eligible households, older children were significantly less likely to take up FSMs. Qualitative research suggests this may reflect stigma, dissatisfaction with food quality, or a preference to leave school premises at lunchtime.^66,67^ Households without a disabled member were also less likely to take up FSMs, potentially because families with disabled members are more connected with support services that help secure entitlements.

Looking to post-September 2026, our projections suggest that extending FSMs to all UC households could expand eligibility for over 270 000 food-insecure children. Yet, more than 610 000 may remain ineligible, and around 81 000 could lose their entitlement due to the ending of legacy benefits and transitional protections. Overall, 44.5% of all food-insecure children may remain or become ineligible. However, it is important to note that these are crude projections, which assume that household circumstances remain unchanged between the survey period and policy implementation in September 2026, including household composition, income and eligibility status.

These patterns were also observed when using a more restrictive classification of food insecurity in sensitivity analyses, with 37.9% of children in households experiencing low or very low food security projected to remain or become ineligible under 2026 changes. This indicates that the group of children not reached by FSM eligibility is not confined to those experiencing marginal food insecurity and highlights that further expansions are necessary to ensure that all food-insecure children are eligible for FSMs.

Crucially, eligibility does not guarantee uptake. If current patterns persist, around one in five eligible children in 2026, may still miss out. Without a dual approach that expands eligibility while also removing barriers to uptake, many vulnerable children may remain unsupported.

An unexpected additional finding was that 42.8% (95% CI 38.9% to 46.7%) of children reported as receiving FSMs were classified as ineligible under the benefit receipt and income criteria applied in this study. Some of this discrepancy may be due to localised extensions of FSM eligibility, income volatility or other eligibility criteria unable to be identified in the FRS, but these are unlikely to be a major source of misclassification. The more likely explanation is transitional protections, which have meant households have remained eligible for FSMs, even if they no longer met benefit receipt and income thresholds. This could be, for example, households who receive UC but whose incomes have risen above income thresholds. We observed that among children in this group, over 60% were in households receiving UC but which had incomes above current thresholds. Given the risks of food insecurity among households receiving UC, this has likely been an important policy.^68^ No national data on FSM receipt through transitional protections are available, but both the Department for Education and Institute for Fiscal Studies have identified transitional protections as a reason for rising numbers of eligible children.^69,70^

### Strengths and limitations

This is the first study to quantify FSM reach for food-insecure children in England using nationally representative data, identifying both drivers of ineligibility and barriers to uptake. However, some limitations should be noted.

FSM receipt was based on the primary carers’ report over the previous week, which may underestimate uptake if the child was absent. Although August was excluded, data collected during other school holidays may also affect accuracy.

Limitations arise from deriving eligibility from survey-based income and benefit data, rather than direct measures of eligibility. Some children received FSMs despite appearing ineligible by application of benefit and income threshold criteria. As above, transitional protections are a likely explanation, as households eligible by these criteria could not be identified. We cannot verify this, however, as no data on the proportion of children receiving FSMs under transitional protections are published. Eligibility under Part VI of the Immigration and Asylum Act 1999 also could not be identified; however, asylum-seeking households are less likely to be sampled in the FRS since they are often not residents in private accommodation.^71,72^ Some misclassification may also arise from localised extensions of FSM eligibility, though this is not common. Finally, converting weekly earnings into annual income may misrepresent eligibility among households with irregular or seasonal earnings. Misclassification of eligibility would make more children appear ineligible among those receiving FSMs and may underestimate non-uptake among food-insecure children not receiving FSMs. Nevertheless, the central finding that a substantial proportion of food-insecure children are not receiving FSMs is robust to this potential misclassification, as this estimate is based on observed FSM receipt rather than derived eligibility status.

Finally, the scenario-based projections of the 2026 eligibility changes are subject to uncertainty. We modelled two plausible scenarios for transition from legacy benefits to UC using available DWP figures; however, these figures are largely derived from tax credit claimant behaviour and may not generalise to other legacy benefit groups.^51^ Results should be interpreted as scenario-based projections rather than precise predictions and are contingent on assumptions that household circumstances remain unchanged between the survey period and policy implementation, and that future transitions from legacy benefits to UC follow the rate applied in this study.

### Further research

Further research should explore, through qualitative methods, the barriers that prevent eligible children from taking up FSMs, particularly those with characteristics linked to higher non-uptake in this study. Evaluation work is needed to track changes in eligibility, uptake and food insecurity, especially as new policies, such as universal FSMs in London and Wales, are implemented. Research with families transitioning from legacy benefits to UC would also help inform the design of support to minimise non-transition to UC.

It is of note that some local authorities have implemented auto-enrolment approaches for FSMs, with wider piloting occurring since 2024.^31^ While this practice was too new in 2022–2023 and 2023–2024 and is therefore unlikely to be reflected in the present analysis, the lack of local area identifiers in the publicly available data precluded examination of uptake in areas where auto-enrolment has been implemented. An important area of future research is to understand how auto-enrolment may reduce disparities in uptake among eligible households, particularly those related to child age and household disability status.^31^

### Policy implications

Our findings have important policy implications and are particularly relevant in the context of the government’s renewed focus on creating the ‘healthiest generation of children ever’ through the Child Poverty Taskforce.^73^

First, the Government’s decision to extend FSM eligibility to all children in UC-recipient households from September 2026 is an important step forward, and our analysis suggests that more than 270 000 additional food-insecure children could become newly eligible as a result. However, 44.5% of all food-insecure children may remain or become ineligible, even after this change. Further eligibility expansion is therefore needed. Importantly, structural inequalities, such as ethnicity, disability and lower educational attainment in the household, remained independently associated with food insecurity after adjustment for current and future eligibility. This highlights how eligibility criteria based on income and benefits do not fully capture children experiencing food insecurity. Without further reforms, large numbers of food-insecure children will continue to be left without access to FSMs.

Second, the closure of legacy benefits and ending of transitional protections also poses a risk of exclusion. DWP figures show that nearly a third of households invited to move to UC do not successfully do so, possibly due to a fear of increased stigma or the complexity of applications.^51^ Our projections suggest that, depending on transition rates, 5% of food-insecure children could lose FSM entitlement. Support strategies to help with this transition are recommended.

Third, low FSM uptake levels among eligible children should also be addressed. One evidence-based approach is national auto-enrolment of eligible children, which has been shown to increase FSM uptake.^74–77^ By removing the need for families to self-determine eligibility or complete applications, this approach could reduce administrative barriers and normalise participation, potentially addressing inequalities in uptake. Additionally, as recommended by Child Poverty Action Group, offering takeaway options, anonymising FSM receipt and involving pupils in menu design could increase participation among older children.^66^ Without focused efforts to increase uptake, around one in five of those eligible post-2026 may still not take up FSMs. Non-uptake among eligible pupils also has wider implications for schools through reduced pupil premium funding that is linked to FSMs.^78^

Ultimately, however, the wide-ranging and complex reasons food-insecure children do not receive FSMs suggest that policies relying on means-tested eligibility and application processes may leave some food-insecure children without support. While evaluation of universal provision was outside the scope of this study, our findings are consistent with and indirectly support arguments for universal FSM provision. Wider literature suggests that universal FSMs may remove administrative barriers, ensure support for all food-insecure children and help address some of the structural inequalities, alongside improvements in nutrition and reduced stigma.^19–23,26–29^ Although implementation costs would be substantial, an independent cost–benefit analysis estimated that every £1 invested in universal FSMs yields £1.71 in returns over 20 years through gains in education, health and productivity.^79^ This return exceeds that estimated from extending FSMs to all families in receipt of UC.^79^

## Conclusion

Between 2022 and 2024, over two thirds of food-insecure children, equivalent to more than 1.1 million food-insecure children each year, went without FSMs. Most appeared ineligible by income and benefits criteria, while around one in five were eligible but did not take up their entitlement. Determining FSM eligibility based on benefit receipt and income thresholds is insufficient for reaching children vulnerable to food insecurity due to persistent structural inequalities. Although the September 2026 eligibility change will extend support to more children, inequities will persist, with 44.5% of food-insecure children estimated to remain or become ineligible. While interim measures, such as national auto-enrolment, are likely to improve access, universal FSM provision is likely a more equitable and effective solution to address both restrictive eligibility and barriers to uptake.

## Supplementary material

10.1136/bmjph-2025-004455 online supplemental file 1

## Data Availability

Data are available in a public, open access repository.
