# Supplementary material for: Do children in food-insecure households in England access free school meals? A cross-sectional analysis of the UK Family Resources Survey
Source: BMJ Public Health. 2026 Sep 18;4(3):e004455. doi: 10.1136/bmjph-2025-004455 (PMC13599944; doi:10.1136/bmjph-2025-004455)
Supplement: Supplementary file 1 [file bmjph-4-3-s001.pdf]

Appendix 1: Flowchart showing derivation of Free School Meal eligibility

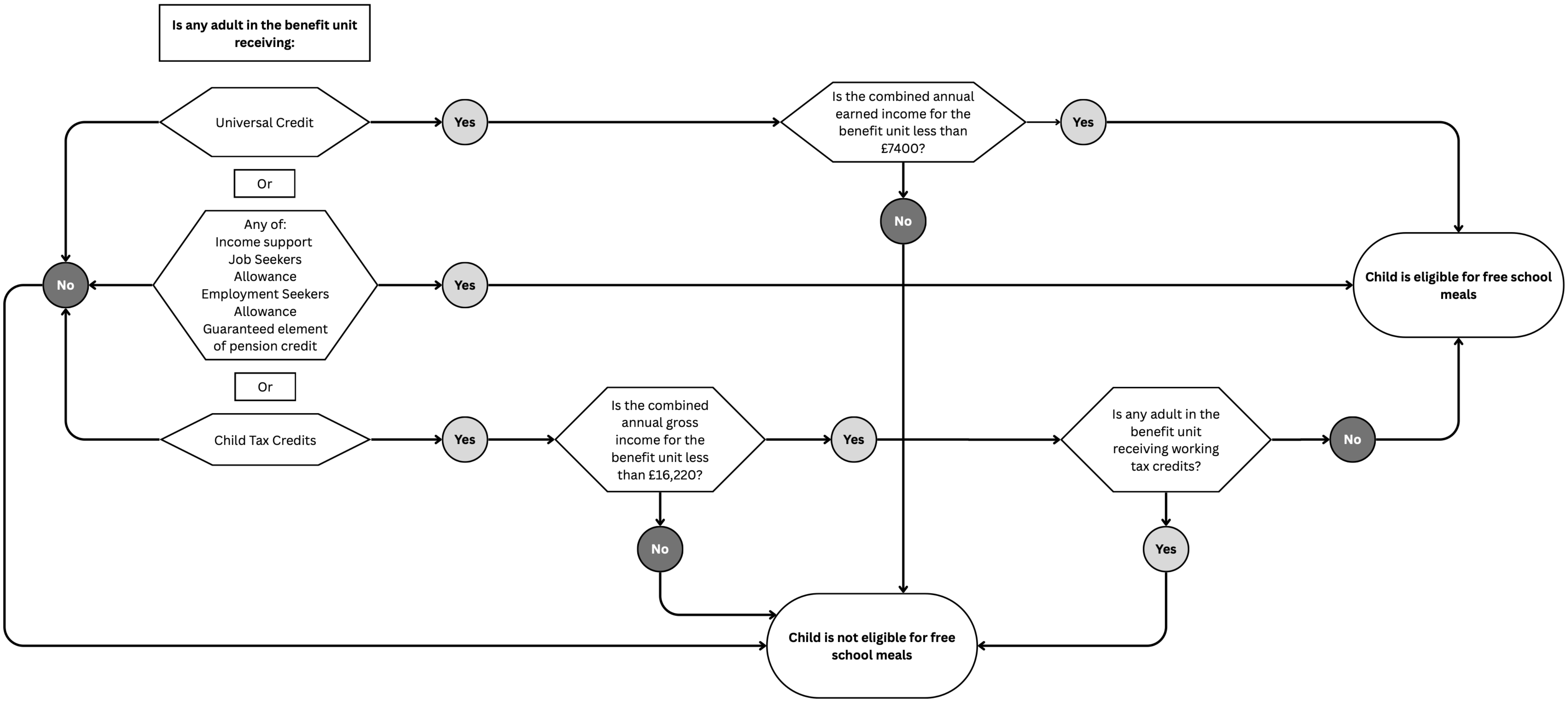

Appendix 2: Flowchart of the analytical sample.

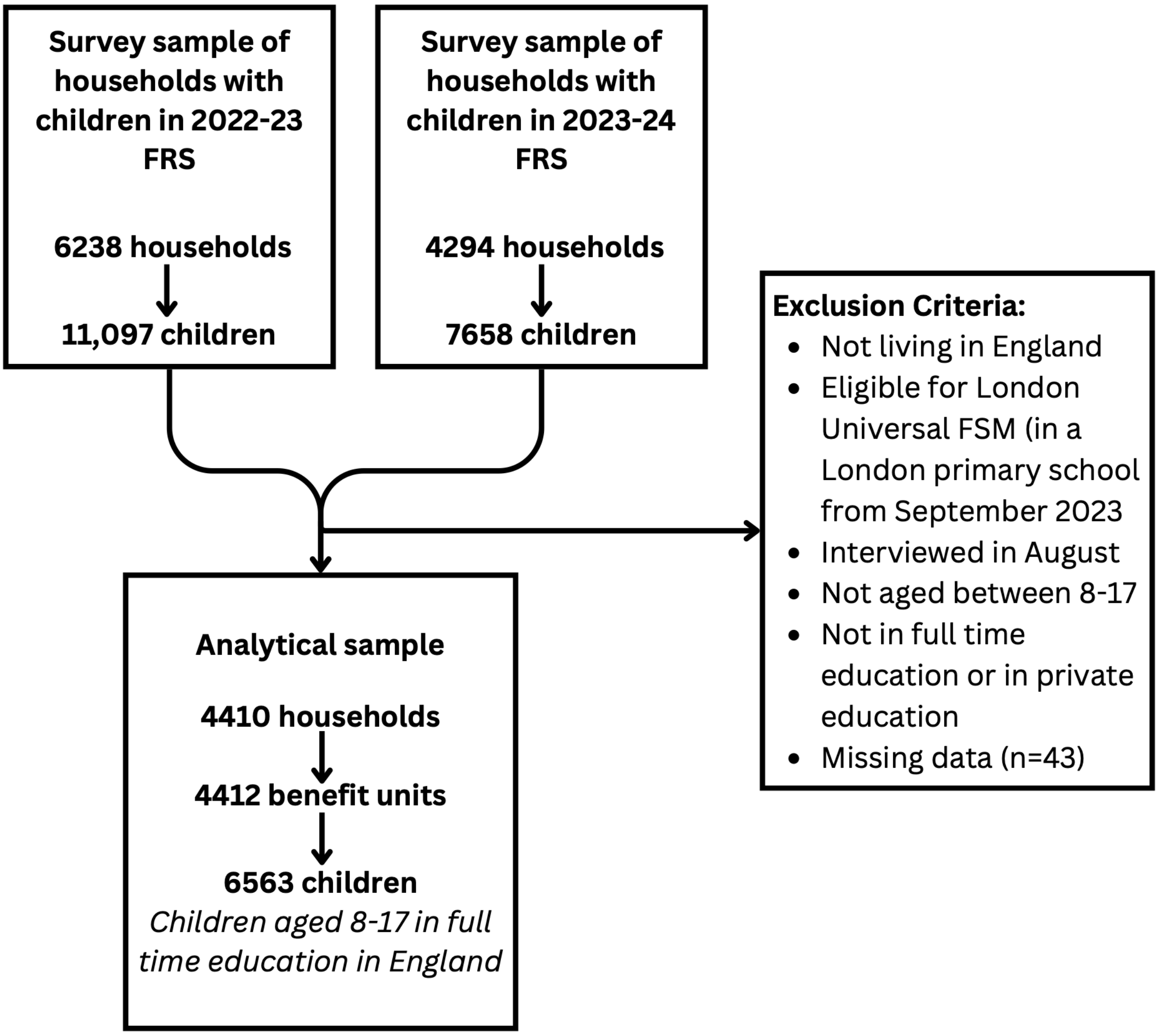

Notes: There were 2 households where children were part of a different benefit unit, hence the numbers of households and benefit units do not match each other.

Appendix 3: Flowchart illustrating the distribution of food insecurity, FSM receipt and eligibility across the population sample between 2022 and 2024, averaged to produce yearly estimates. With accompanying confidence intervals.

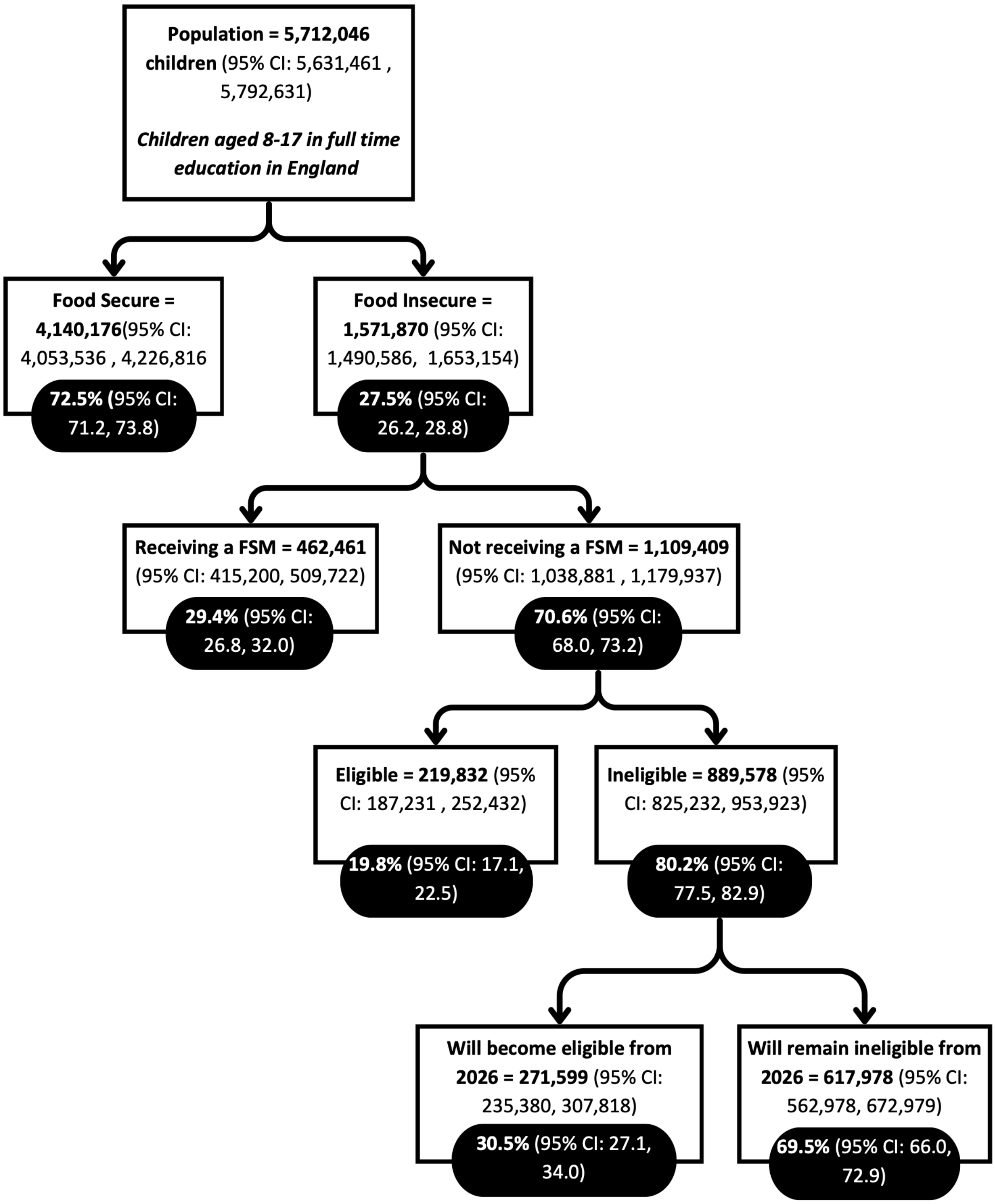

Notes: Eligibility was classified using the derived eligibility criteria described in the Methods, rather than observed administrative eligibility

Appendix 4: Odds of food insecurity among households where at least one child is not receiving FSMs (n=3784).

	Odds of Food Insecurity (OR (Lower CI, Upper CI))			
	**Base Model** (Food Insecurity ~ FSM eligibility)	**Model 1:** Base Model + Universal Credit and Legacy Benefits	**Model 2:** Base Model + Universal Credit and Legacy Benefits + other benefits	**Model 3:** Base Model + all benefits + other predictors
**Current FSM eligibility**[1]				
Not eligible	Reference Category	Reference Category	Reference Category	Reference Category
Eligible (based on benefit receipt / income)	**5.97 (4.65, 7.65)**	**1.86 (1.42, 2.44)**	**1.74 (1.3, 2.31)**	1.21 (0.75, 1.95)
**Receipt of benefits**				
No benefits	---	Reference Category	Reference Category	Reference Category
Legacy benefits[2]		**5.72 (4.54, 7.2)**	**5.6 (4.42, 7.1)**	**2.49 (1.86, 3.35)**
Universal Credit		**6.27 (5.08, 7.75)**	**6.22 (5, 7.74)**	**2.78 (2.11, 3.66)**
**Receipt of Disability Benefits**[3]				
Not in receipt	---	---	Reference Category	Reference Category
In receipt			1.31 (0.93, 1.86)	1.19 (0.82, 1.73)
**Receipt of Child Benefits**				
Not in receipt	---	---	Reference Category	Reference Category
In receipt			1.02 (0.77, 1.35)	1.02 (0.75, 1.38)
**Annual earned family income**				
Under income threshold	---	---	---	Reference Category
£7400 - £10,999				1.27 (0.74, 2.18)
£11,000 to £14,999				0.89 (0.53, 1.48)
£15,000 to £25,999				0.83 (0.51, 1.34)
£26,000 to £39,999				0.92 (0.56, 1.52)
£40,000 to £66,199				0.62 (0.37, 1.03)
£66,200 or higher				**0.24 (0.13, 0.44)**

**Ethnicity of first parent**				
White	---	---	---	Reference Category
Asian/ Asian British				1.28 (0.95, 1.72)
Black/ African/ Caribbean/ Black British				**2.37 (1.64, 3.42)**
Mixed/ Multiple ethnic groups				0.86 (0.45, 1.66)
Other ethnic group				1.35 (0.78, 2.32)
**Highest education level in household**				
Higher degree of postgraduate qualifications	---	---	---	Reference Category
Undergraduate Degree and equivalent				1.31 (0.97, 1.79)
Diplomas in higher education and equivalent				**1.9 (1.32, 2.75)**
A/AS levels and equivalent				**2.04 (1.48, 2.83)**
O level / GCSE grades A-C and equivalent				**2.33 (1.61, 3.38)**
O level / GCSE grades D-G and equivalent				**2.43 (1.61, 3.67)**
No answer provided				**3.1 (2.01, 4.79)**
**Disabled individuals in household**				
No disabled individuals	---	---	---	Reference Category
At least one disabled individual				**1.71 (1.41, 2.06)**
**Household full-time employment**				
No individuals in full-time employment	---	---	---	Reference Category
At least one individual in full-time employment				0.97 (0.72, 1.3)

**Unemployment in household**				
No individuals unemployed	---	---	---	Reference Category
At least one individual unemployed				**2.01 (1.3, 3.08)**
**Lone parent household**				
No	---	---	---	Reference Category
Yes				**1.33 (1.05, 1.69)**
**Region**				
South				Reference Category
London	---	---	---	**1.37 (1.02, 1.83)**
North				1.08 (0.88, 1.31)
**Number of children, *per additional child***	---	---	---	**1.11 (1, 1.24)**
**Children in Primary School**				
No children in household in primary school	---	---	---	Reference Category
At least one child in household in primary school				1.18 (0.92, 1.51)
**Children in Secondary School**				
No children in household in secondary school	---	---	---	Reference Category
At least one child in household in secondary school				1.07 (0.82, 1.39)

Notes*:* (1) Eligibility was classified using the derived eligibility criteria described in the Methods, rather than observed administrative eligibility (2) Legacy benefits include housing benefits; working tax credits, child tax credits, income support, job seekers allowance, employment and support allowance and pension credit (3) Disability Benefits include Personal Independence Payment, Adult Disability Payment, disability living allowance or child disability payment. Bold font indicates results that are statistically significant ($p < 0.05$).

Appendix 5: Proportionate Sankey diagram showing the forecasted change in FSM reach among children living in food-insecure households (n=1,571,870) following eligibility changes from September 2026 under Scenario 1 (a) and Scenario 2 (b). Averaged to produce yearly estimates. Percentages represent proportion of all children in food-insecure households present in each category. With accompanying confidence intervals

Notes: Numbers indicate yearly estimates. Two scenarios were modelled to forecast the effect of the closure of legacy benefits on FSM eligibility. Scenario 1: All households successfully transition to UC and remain eligible. Scenario 2: 27% of households on legacy benefits do not transition to UC (DWP statistics) [51]. Both scenarios are conditional on assumptions laid out in the method and should be interpreted as scenario-based projections rather than predictions. Current eligibility was classified using the derived eligibility criteria described in the Methods, rather than observed administrative eligibility. To see these diagrams without confidence intervals, please see figure 2 and 3.

a) Scenario 1

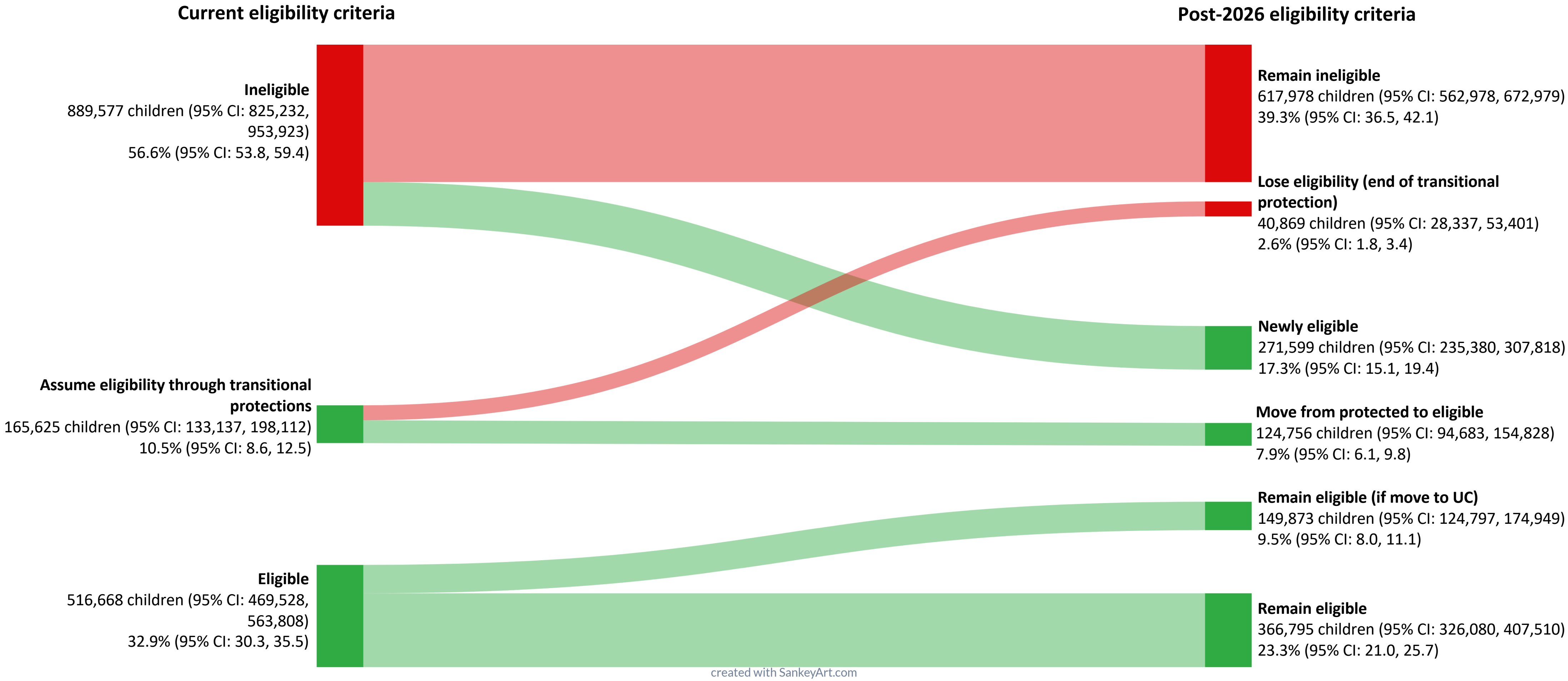

Current eligibility criteria
Post-2026 eligibility criteria
Ineligible
889,577 children (95% CI: 825,232, 953,923)
56.6% (95% CI: 53.8, 59.4)
Assume eligibility through transitional protections
165,625 children (95% CI: 133,137, 198,112)
10.5% (95% CI: 8.6, 12.5)
Eligible
516,668 children (95% CI: 469,528, 563,808)
32.9% (95% CI: 30.3, 35.5)
Remain ineligible
617,978 children (95% CI: 562,978, 672,979)
39.3% (95% CI: 36.5, 42.1)
Lose eligibility (end of transitional protection)
40,869 children (95% CI: 28,337, 53,401)
2.6% (95% CI: 1.8, 3.4)
Newly eligible
271,599 children (95% CI: 235,380, 307,818)
17.3% (95% CI: 15.1, 19.4)
Move from protected to eligible
124,756 children (95% CI: 94,683, 154,828)
7.9% (95% CI: 6.1, 9.8)
Remain eligible (if move to UC)
149,873 children (95% CI: 124,797, 174,949)
9.5% (95% CI: 8.0, 11.1)
Remain eligible
366,795 children (95% CI: 326,080, 407,510)
23.3% (95% CI: 21.0, 25.7)
created with SankeyArt.com

b) Scenario 2

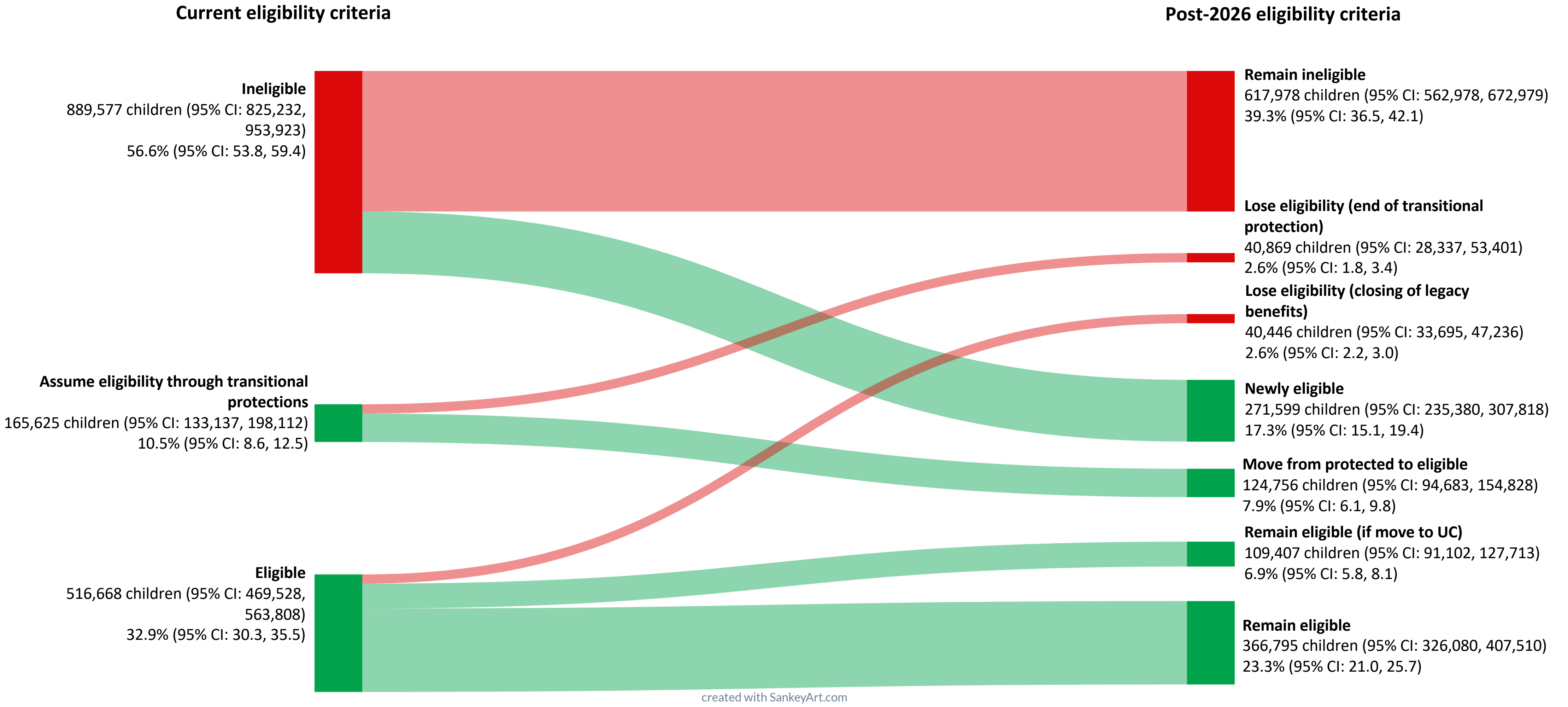
Current eligibility criteria
Post-2026 eligibility criteria
Ineligible
889,577 children (95% CI: 825,232, 953,923)
56.6% (95% CI: 53.8, 59.4)
Assume eligibility through transitional protections
165,625 children (95% CI: 133,137, 198,112)
10.5% (95% CI: 8.6, 12.5)
Eligible
516,668 children (95% CI: 469,528, 563,808)
32.9% (95% CI: 30.3, 35.5)
Remain ineligible
617,978 children (95% CI: 562,978, 672,979)
39.3% (95% CI: 36.5, 42.1)
Lose eligibility (end of transitional protection)
40,869 children (95% CI: 28,337, 53,401)
2.6% (95% CI: 1.8, 3.4)
Lose eligibility (closing of legacy benefits)
40,446 children (95% CI: 33,695, 47,236)
2.6% (95% CI: 2.2, 3.0)
Newly eligible
271,599 children (95% CI: 235,380, 307,818)
17.3% (95% CI: 15.1, 19.4)
Move from protected to eligible
124,756 children (95% CI: 94,683, 154,828)
7.9% (95% CI: 6.1, 9.8)
Remain eligible (if move to UC)
109,407 children (95% CI: 91,102, 127,713)
6.9% (95% CI: 5.8, 8.1)
Remain eligible
366,795 children (95% CI: 326,080, 407,510)
23.3% (95% CI: 21.0, 25.7)
created with SankeyArt.com

Appendix 6: Flowchart illustrating the distribution of low or very low food security, FSM receipt and eligibility across the population sample between 2022 and 2024, averaged to produce yearly estimates. With accompanying confidence intervals

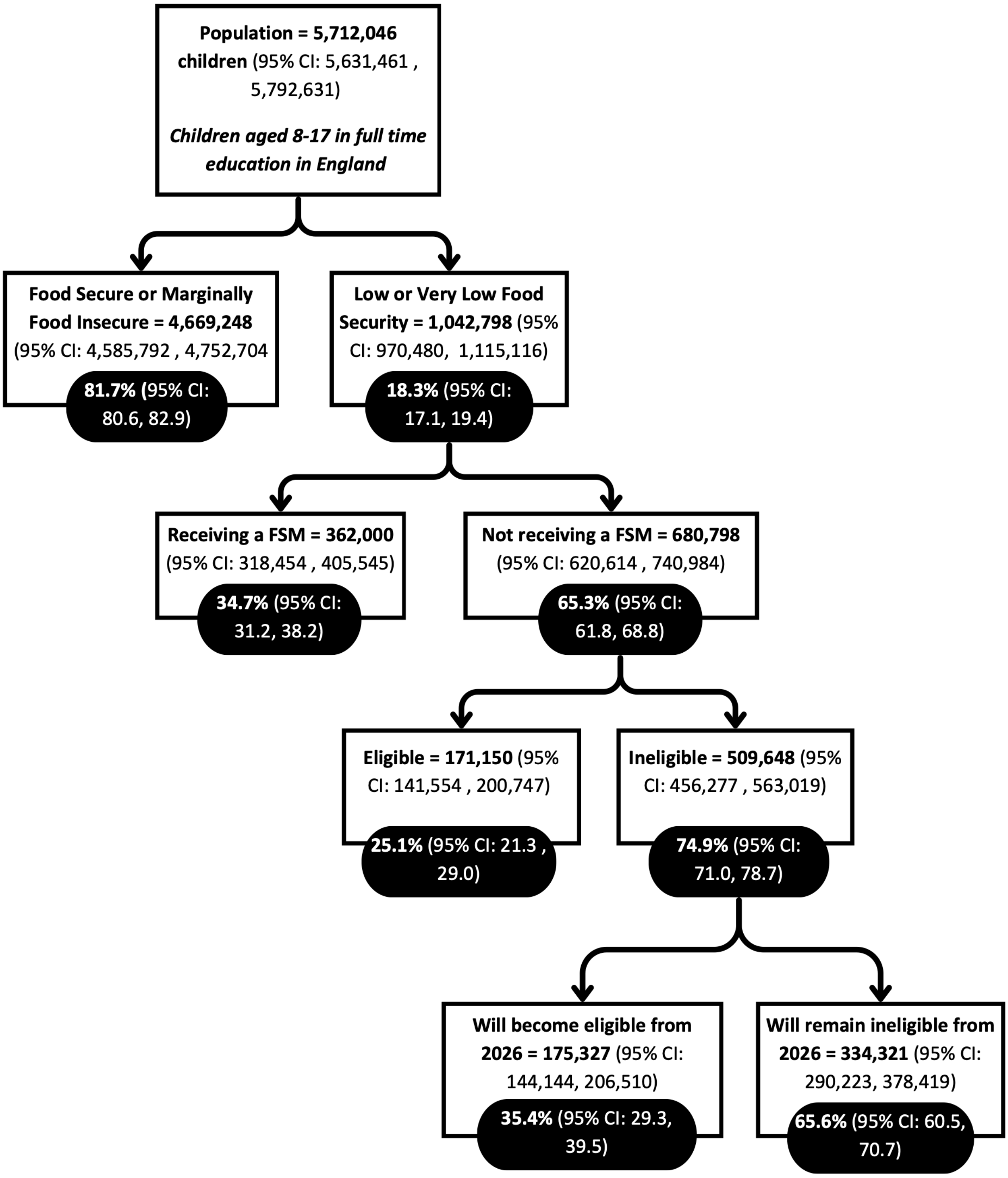

Notes: Eligibility was classified using the derived eligibility criteria described in the Methods, rather than observed administrative eligibility

Appendix 7: Sensitivity analysis for logistic regression models calculating odds of low or very low food security amongst households with at least one child not receiving FSMs (n=3784).

	**Odds of Low or Very Low Food Security (OR (Lower CI, Upper CI))**			
	**Base Model** (Food Insecurity ~ FSM eligibility)	**Model 1:** Base Model + Universal Credit and Legacy Benefits	**Model 2:** Base Model + Universal Credit and Legacy Benefits + other benefits	**Model 3:** Base Model + all benefits + other predictors
**Current FSM eligibility[1]**				
Not eligible	Reference Category	Reference Category	Reference Category	Reference Category
Eligible (based on benefit receipt / income)	**6.87 (5.31, 8.91)**	**2.32 (1.75, 3.08)**	**2.17 (1.6, 2.93)**	1.68 (0.97, 2.91)
**Receipt of benefits**				
No benefits	---	Reference Category	Reference Category	Reference Category
Legacy benefits[1]		**5.91 (4.47, 7.83)**	**5.87 (4.39, 7.86)**	**1.97 (1.37, 2.83)**
Universal Credit		**6.25 (4.82, 8.12)**	**6.3 (4.81, 8.27)**	**2.2 (1.57, 3.09)**
**Receipt of Disability Benefits[3]**				
Not in receipt	---	---	Reference Category	Reference Category
In receipt			1.28 (0.88, 1.87)	1.05 (0.7, 1.56)
**Receipt of Child Benefits**				
Not in receipt	---	---	Reference Category	Reference Category
In receipt			0.94 (0.65, 1.36)	0.9 (0.61, 1.33)
**Annual earned family income**				
Under income threshold	---	---	---	Reference Category
£7400 - £10,999				1.61 (0.88, 2.96)
£11,000 to £14,999				1.03 (0.57, 1.87)
£15,000 to £25,999				0.95 (0.54, 1.67)
£26,000 to £39,999				0.93 (0.51, 1.69)
£40,000 to £66,199				**0.47 (0.25, 0.87)**

£66,200 or higher				**0.18 (0.08, 0.41)**
**Ethnicity of first parent**				
White	---	---	---	Reference Category
Asian/ Asian British				0.96 (0.66, 1.4)
Black/ African/ Caribbean/ Black British				**2.35 (1.54, 3.6)**
Mixed/ Multiple ethnic groups				0.98 (0.46, 2.1)
Other ethnic group				1.53 (0.82, 2.83)
**Highest education level in household**				
Higher degree of postgraduate qualifications	---	---	---	Reference Category
Undergraduate Degree and equivalent				1.46 (0.95, 2.23)
Diplomas in higher education and equivalent				**1.88 (1.14, 3.07)**
A/AS levels and equivalent				**2.23 (1.44, 3.44)**
O level / GCSE grades A-C and equivalent				**2.8 (1.75, 4.51)**
O level / GCSE grades D-G and equivalent				**2.85 (1.71, 4.74)**
No answer provided				**3.3 (1.95, 5.57)**
**Disabled individuals in household**				
No disabled individuals	---	---	---	Reference Category
At least one disabled individual				**1.71 (1.41, 2.06)**
**Household full-time employment**				
No individuals in full-time employment	---	---	---	Reference Category
At least one individual in full-time employment				0.97 (0.69, 1.37)
**Unemployment in household**				
No individuals unemployed	---	---	---	Reference Category
At least one individual unemployed				1.23 (0.75, 2.02)
**Lone parent household**	---	---	---	
No				Reference Category

Yes				**1.27 (0.96, 1.67)**
**Region**				
South	---	---	---	Reference Category
London				1.37 (0.96, 1.95)
North				1.15 (0.91, 1.47)
**Number of children, *per additional child***	---	---	---	**1.2 (1.06, 1.36)**
**Children in Primary School**				
No children in household in primary school	---	---	---	Reference Category
At least one child in household in primary school				1.09 (0.82, 1.47)
**Children in Secondary School**				
No children in household in secondary school	---	---	---	Reference Category
At least one child in household in secondary school				1.16 (0.84, 1.6)

***Notes**:* (1) Eligibility was classified using the derived eligibility criteria described in the Methods, rather than observed administrative eligibility (2) Legacy benefits include housing benefits; working tax credits, child tax credits, income support, job seekers allowance, employment and support allowance and pension credit (3) Disability Benefits include Personal Independence Payment, Adult Disability Payment, disability living allowance or child disability payment. Bold font indicates results that are statistically significant ($p < 0.05$).

Appendix 8: Sensitivity analysis for logistic regression models calculating odds of not receiving FSMs amongst eligible[1] children living in households with low or very low food security (n=428).

	**Odds of not receiving Free School Meals (OR (Lower CI, Upper CI)**	
	**Bivariate Model**	**Multivariate Model**
**Age, *per year increase***	**1.16 (1.08, 1.25)**	**1.19 (1.1, 1.29)**
**Region**		
South	Reference Category	Reference Category
London	0.89 (0.49, 1.61)	0.98 (0.48, 1.98)
North	0.72 (0.47, 1.09)	0.67 (0.42, 1.06)
**Lone parent household**		
No	Reference Category	Reference Category
Yes	0.9 (0.59, 1.37)	1.06 (0.64, 1.78)
**Ethnicity of first parent**		
White ethnicity	Reference Category	Reference Category
Minoritised ethnicity	0.75 (0.46, 1.23)	**0.45 (0.23, 0.84)**
**Receipt of benefits**		
Legacy benefits[2]	Reference Category	Reference Category
Universal Credit	0.99 (0.65, 1.51)	1.12 (0.69, 1.85)
**Receipt of Disability Benefits[3]**		
Not in receipt	Reference Category	Reference Category
In receipt	1.25 (0.83, 1.88)	1.62 (0.96, 2.72)
**Receipt of Child Benefits**		
Not in receipt	Reference Category	Reference Category
In receipt	0.5 (0.16, 1.47)	0.58 (0.17, 1.87)
**Number of children*, per additional child***	0.89 (0.77, 1.04)	1 (0.84, 1.19)
**Highest education level in household**		
Undergraduate or higher degree of postgraduate qualifications	1.97 (0.96, 4.08)	2.09 (0.96, 4.66)
Diplomas in higher education and equivalent	1.38 (0.62, 3.06)	1.33 (0.56, 3.16)
A/AS levels and equivalent	Reference Category	Reference Category
O level / GCSE and equivalent	0.77 (0.46, 1.28)	0.72 (0.41, 1.24)

Not Answered/Don't know/Prefer not to say	1.45 (0.81, 2.61)	1.45 (0.77, 2.75)
**Disabled individuals in household**		
No disabled individuals	Reference Category	Reference Category
At least one disabled individual	**0.44 (0.26, 0.73)**	**0.27 (0.15, 0.5)**
**Household full-time employment**		
No individuals in full-time employment	Reference Category	Reference Category
At least one individual in full-time employment	1.51 (0.76, 3.01)	1.16 (0.52, 2.6)
**Unemployment in household**		
No individuals unemployed	Reference Category	Reference Category
At least one individual unemployed	1.16 (0.6, 2.21)	1.02 (0.47, 2.14)

Notes*:* (1) Eligibility was classified using the derived eligibility criteria described in the Methods, rather than observed administrative eligibility (2) Legacy benefits include housing benefits; working tax credits, child tax credits, income support, job seekers allowance, employment and support allowance and pension credit (3) Disability Benefits includes Personal Independence Payment, Adult Disability Payment, disability living allowance or child

Appendix 9: Sensitivity analysis results. Proportionate Sankey diagram showing the forecasted change in FSM reach among children living in households reporting low or very low food security (n=1,042,797) following eligibility changes from September 2026 under Scenario 1 (a) and Scenario 2 (b). Averaged to produce yearly estimates. Percentages represent proportion of all children in food-insecure households present in each category. With accompanying confidence intervals

Notes: Numbers indicate yearly estimates. Two scenarios were modelled to forecast the effect of the closure of legacy benefits on FSM eligibility. Scenario 1: All households successfully transition to UC and remain eligible. Scenario 2: 27% of households on legacy benefits do not transition to UC (DWP statistics) [51]. Both scenarios are conditional on assumptions laid out in the method and should be interpreted as scenario-based projections rather than predictions. Current eligibility was classified using the derived eligibility criteria described in the Methods, rather than observed administrative eligibility.

a) Scenario 1

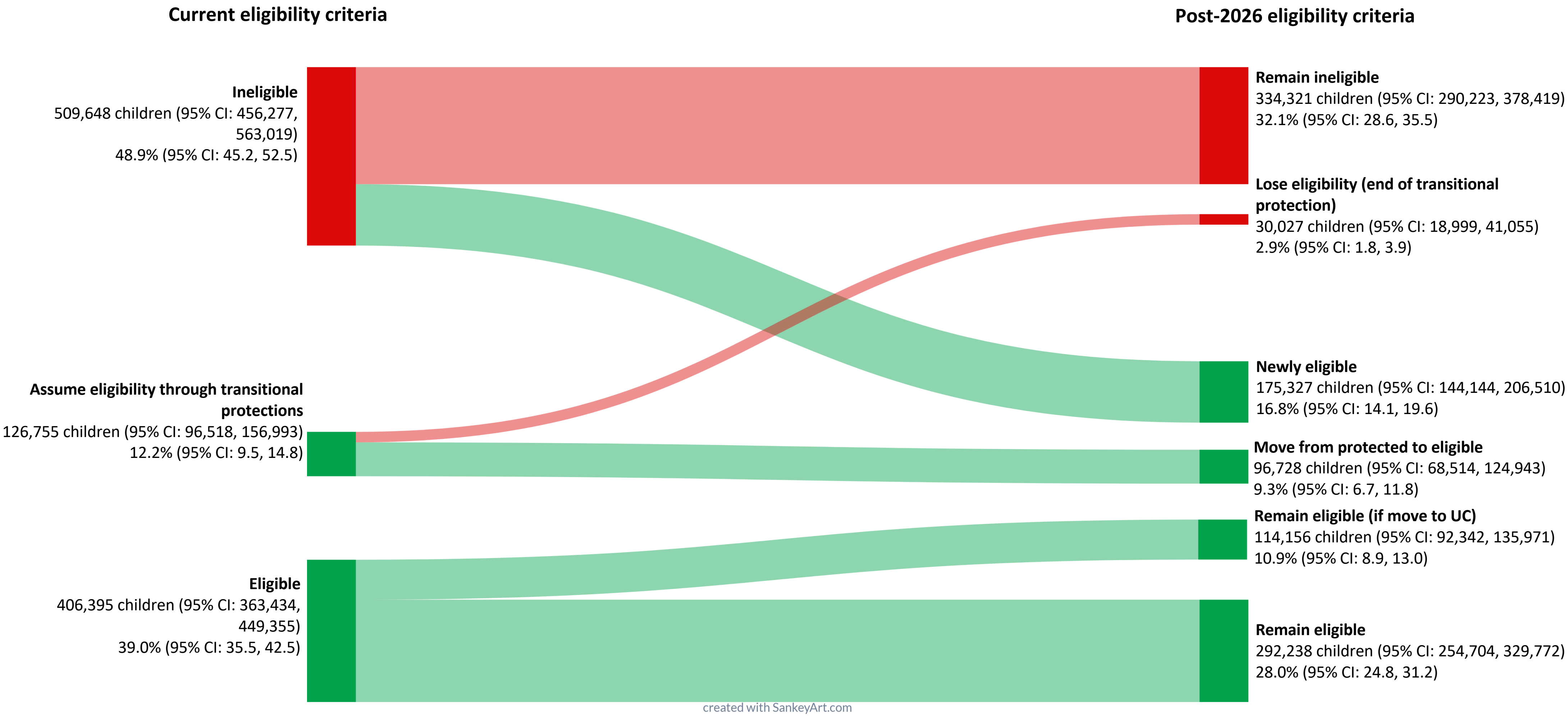
Current eligibility criteria
Post-2026 eligibility criteria
Ineligible
509,648 children (95% CI: 456,277, 563,019)
48.9% (95% CI: 45.2, 52.5)
Assume eligibility through transitional protections
126,755 children (95% CI: 96,518, 156,993)
12.2% (95% CI: 9.5, 14.8)
Eligible
406,395 children (95% CI: 363,434, 449,355)
39.0% (95% CI: 35.5, 42.5)
Remain ineligible
334,321 children (95% CI: 290,223, 378,419)
32.1% (95% CI: 28.6, 35.5)
Lose eligibility (end of transitional protection)
30,027 children (95% CI: 18,999, 41,055)
2.9% (95% CI: 1.8, 3.9)
Newly eligible
175,327 children (95% CI: 144,144, 206,510)
16.8% (95% CI: 14.1, 19.6)
Move from protected to eligible
96,728 children (95% CI: 68,514, 124,943)
9.3% (95% CI: 6.7, 11.8)
Remain eligible (if move to UC)
114,156 children (95% CI: 92,342, 135,971)
10.9% (95% CI: 8.9, 13.0)
Remain eligible
292,238 children (95% CI: 254,704, 329,772)
28.0% (95% CI: 24.8, 31.2)
created with SankeyArt.com

b) Scenario 2

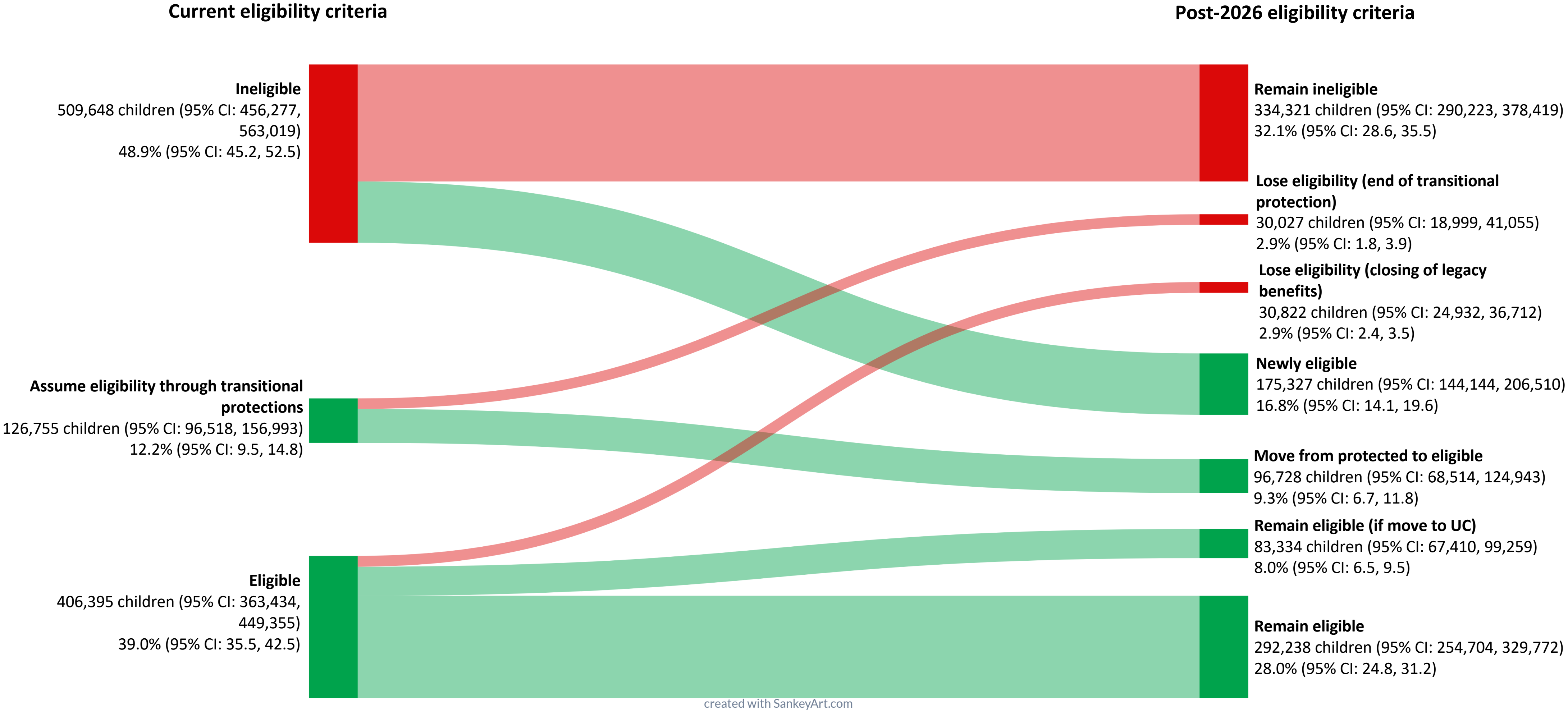
Current eligibility criteria
Post-2026 eligibility criteria
Ineligible
509,648 children (95% CI: 456,277, 563,019)
48.9% (95% CI: 45.2, 52.5)
Assume eligibility through transitional protections
126,755 children (95% CI: 96,518, 156,993)
12.2% (95% CI: 9.5, 14.8)
Eligible
406,395 children (95% CI: 363,434, 449,355)
39.0% (95% CI: 35.5, 42.5)
Remain ineligible
334,321 children (95% CI: 290,223, 378,419)
32.1% (95% CI: 28.6, 35.5)
Lose eligibility (end of transitional protection)
30,027 children (95% CI: 18,999, 41,055)
2.9% (95% CI: 1.8, 3.9)
Lose eligibility (closing of legacy benefits)
30,822 children (95% CI: 24,932, 36,712)
2.9% (95% CI: 2.4, 3.5)
Newly eligible
175,327 children (95% CI: 144,144, 206,510)
16.8% (95% CI: 14.1, 19.6)
Move from protected to eligible
96,728 children (95% CI: 68,514, 124,943)
9.3% (95% CI: 6.7, 11.8)
Remain eligible (if move to UC)
83,334 children (95% CI: 67,410, 99,259)
8.0% (95% CI: 6.5, 9.5)
Remain eligible
292,238 children (95% CI: 254,704, 329,772)
28.0% (95% CI: 24.8, 31.2)
created with SankeyArt.com
